# Métastases cérébrales d’un léiomyosarcome utérin

**DOI:** 10.11604/pamj.2018.30.90.15570

**Published:** 2018-05-31

**Authors:** Hafsa Chahdi, Mohamed Oukabli

**Affiliations:** 1 Service d’Anatomie Pathologique, Hôpital Militaire d’Instruction Mohamed V Rabat, Maroc

**Keywords:** Leiomyosarcome utérin, métastases, cerveau, Uterine leiomyosarcoma, metastases, brain

## Abstract

We here report the case of a 46-year old female patient with a history of total hysterectomy performed in 2011 due to uterine leiomyosarcoma followed by sessions of radiotherapy, who presented with right hemicorporal sensorimotor deficit of acute onset. Clinical examination showed right pyramidal syndrome. Brain magnetic resonance imaging (MRI) revealed a left frontoparietal extra-axial aggressive tumor (A). The patient underwent surgical resection of the cerebral lesion. Anatomo-pathological examination showed fusocellular tumor proliferation (B). Tumor cells looked like discreetly atypical muscle cells and showed few mitotic figures as well focal necrosis. Tumor proliferation infiltrated the bone, the soft tisuues and the dura-mater. Immunohistochemical examination was performed which showed anti h-caldesmon-positive cells (C) and smooth muscle anti-actin-positive cells. The diagnosis of brain metastasis from leiomyosarcoma was retained. The patient underwent radiotherapy sessions with improvement of the motor deficit.

## Image en médecine

Une patiente de 46 ans, ayant comme antécédents une hystérectomie totale en 2011 pour léiomyosarcome utérin suivie de séances de radiothérapie, a présenté brutalement un déficit hémicorporel droit et chez qui l'examen clinique a trouvé un syndrome pyramidal droit. L'imagerie par résonance magnétique (IRM) cérébrale a montré la présence d'un processus tumoral agressif extra axial fronto-parietal gauche (A). La patiente a bénéficié d'une exérèse chirurgicale de la lésion cérébrale. L'examen anatomopathologique a objectivé l'existence d'une prolifération tumorale d'architecture fusocellulaire (B). Les cellules tumorales sont d'allures musculaires discrètement atypiques, quelques figures de mitose sont notées ainsi que des foyers de nécrose. Cette prolifération tumorale infiltrait l'os, les parties molles et la dure-mère. Une étude immunohistochimique a été réalisé et a objectivé un marquage positif des cellules tumorales par l'anti h-caldesmone (C) et l'anti actine muscle lisse. Le diagnostic de métastase cérébrale d'un leiomyosarcome a été retenu. La patiente a subi des séances de radiothérapie avec comme résultat actuel une amélioration du déficit moteur.

**Figure 1:**
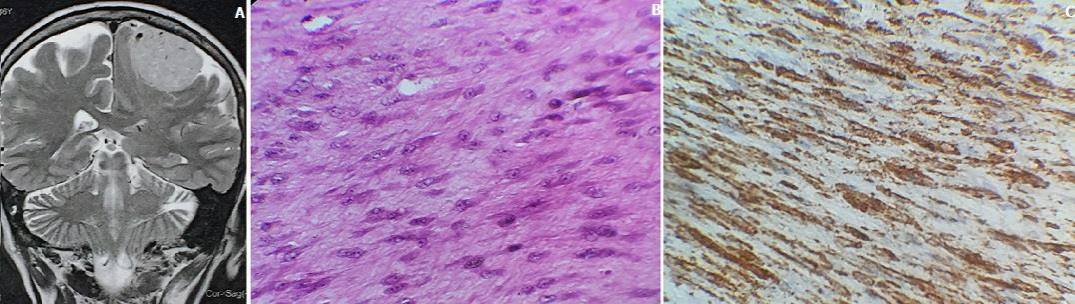
A) IRM, processus tumoral agressif extra axial fronto-parietal gauche; B) aspect microscopique de la prolifération tumorale d’architecture fusocellulaire et d’allure musculaire (Hemateine Eosine GX20); C) immunohistochimie: positivité franche des cellules tumorales par l’anti h-caldesmone

